# Large Retroperitoneal Haemorrhage Following Cyst Rupture in a Patient with Autosomal Dominant Polycystic Kidney Disease

**DOI:** 10.1155/2017/4653267

**Published:** 2017-10-18

**Authors:** Holly Mabillard, Shalabh Srivastava, Philip Haslam, Maciej Karasek, John A. Sayer

**Affiliations:** ^1^Renal Services, Newcastle upon Tyne Hospitals NHS Foundation Trust, Newcastle upon Tyne, UK; ^2^Nephrology Department, Sunderland Royal Hospital, Sunderland, UK; ^3^Interventional Radiology, Newcastle upon Tyne Hospitals NHS Foundation Trust, Newcastle upon Tyne, UK; ^4^Interventional Radiology, Sunderland Royal Hospital, Sunderland, UK; ^5^Institute of Genetic Medicine, Newcastle University, Newcastle upon Tyne, UK

## Abstract

The complications of autosomal dominant polycystic kidney disease (ADPKD) include cyst rupture and haemorrhage leading to loin pain and frank haematuria. Risk factors include large kidney volume, hypertension, and renal impairment. We present a case of a young male who, following trauma to the kidney, had a life threatening bleed from his polycystic kidney. The case was initially treated with fluid resuscitation and blood transfusion but necessitated radiological embolization of bleeding source to control the blood loss. We review the risk factors and management of cyst haemorrhage in patients with ADPKD. Contact sports should be avoided as cyst rupture can lead to severe life threatening haemorrhage.

## 1. Introduction

Autosomal dominant polycystic kidney disease (ADPKD) is characterised by the progressive development and enlargement of kidney cysts throughout the renal tissue, which leads to increases in renal volume and ultimately renal failure. Cysts may also affect other organs, typically the liver. A measurement of height adjusted renal volume allows prediction of progression to end stage renal disease [1]. Haematuria is a common renal manifestation of ADPKD [2]. Haematuria may often be visible and can be the presenting feature of this disease [3]. A urinary tract infection or strenuous activity may precipitate an episode of frank haematuria, and episodes may be recurrent. Haematuria is most often secondary to the rupture of a kidney cyst, which may present with pain alone or pain followed by haematuria. Conservative therapy usually allows resolution of the haematuria over several days however there may bleeding from ruptured cysts that lasts for weeks or is severe enough to warrant intervention with arterial embolization. We present a case of a patient with ADPKD complicated by a life threatening bleed.

## 2. Case Presentation

**Figure 1:**
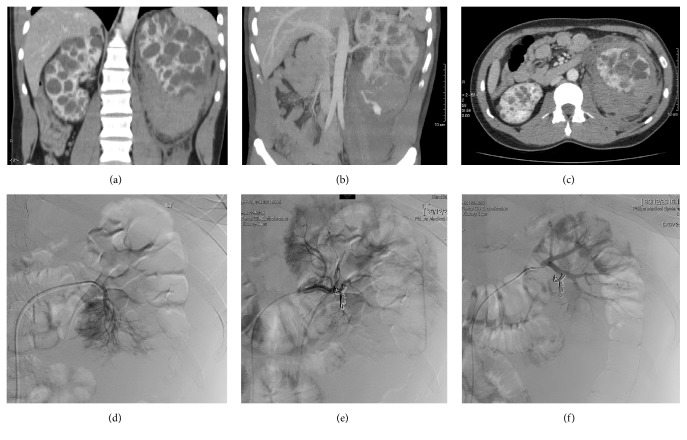
*Computerised tomography (CT) of abdomen and left renal arteriogram and embolization of a patient with autosomal dominant polycystic kidney disease demonstrating large left renal retroperitoneal haemorrhage*. Sagittal views (a and b) and (c) axial view of contrast enhanced CT abdomen showing bilateral polycystic kidneys with evidence of large volume haemorrhage which surrounds the left kidney and extends into the retroperitoneal space. Super-selective angiogram of the left renal artery lower polar segmental branch showing an extravasation into the lower polar renal cyst (d) and final, postembolization angiogram confirming satisfactory exclusion of the source of bleed (e and f).

A 25-year-old male with a known diagnosis of autosomal dominant polycystic kidney disease (ADPKD), with preserved renal function, presented with sudden onset severe left flank, following a rugby game. The loin pain was associated with frank haematuria. On examination, he had severe tenderness to his left hypochondrium and renal angle. He was tachycardic and blood pressure was elevated at an average of 160/90 from a baseline of 130/80. A CT abdomen (Figures 1(a), 1(b), and 1(c)) revealed a large left sided haemorrhage surrounding the polycystic kidney which extended into the retroperitoneum. The renal lengths and volumes were 15.7 cm and 571 mL, respectively, on the right and 16.0 cm and 732 mL, respectively, on the left. He was managed supportively with intravenous fluids (0.9% sodium chloride solution, 125 mLs/h), analgesia (including paracetamol and codeine), and haemodynamic monitoring. Twelve hours after presentation his haemoglobin dropped from 132 g/L to 60 g/L with an associated fall in haematocrit, but with maintenance of blood pressure. This was treated with a blood transfusion (3 units packed red cells) and radiological embolization of the bleeding source (Figures 1(d), 1(e), and 1(f)). Renal function remained stable and the patient improved without the need for further intervention. He was advised to avoid contact sports in future and his haematuria and pain resolved within two weeks.

## 3. Discussion

Up to 70% of patients with ADPKD develop cyst haemorrhage and haematuria due to rupture into the collecting system which is usually self-limiting [4, 5]. The risk of haemorrhage is increased with increasing kidney volume, especially if kidneys are greater than 15 cm in length, as was the case here, or if there is hypertension or renal impairment [3, 6]. Patients with* PKD1* mutations generally have a more severe disease course than patient with* PKD2* mutations, with a larger number of cysts and a faster progression of disease. Episodes of frank haematuria below the age of 30 years have been associated with risk of rapid disease progression [7] and a urological event before the age of 35; for example, frank haematuria contributes to an increase in PROPKD score, a recently developed algorithm to predict renal outcomes in ADPKD [8]. Patients affected with ADPKD should be advised to avoid contact sports (such as boxing, rugby, and American football) because of the associated cyst rupture risk. Useful information regarding lifestyle can be found on the PKD charity website (http://www.pkdcharity.org.uk/).

Haematuria is mostly managed conservatively with intravenous fluids and analgesia. In cases of prolonged bleeding, clot formation can occur causing renal tract obstruction. In this instance, a three-way catheter and bladder irrigation are required and if bleeding persists for over 2 weeks, ureteric stenting may be necessary. In severe bleeding and extensive haematomas, blood transfusion, percutaneous transcatheter renal artery embolization, or even surgical approaches such as nephrectomy may be necessary [9]. Other important differential diagnoses of renal angle tenderness in a patient with ADPKD include cyst infection, cyst enlargement, pyelonephritis, nephrolithiasis, and tumours, including renal cell carcinoma [9].
